# Curdione and Schisandrin C Synergistically Reverse Hepatic Fibrosis via Modulating the TGF-β Pathway and Inhibiting Oxidative Stress

**DOI:** 10.3389/fcell.2021.763864

**Published:** 2021-11-10

**Authors:** Wenzhang Dai, Qin Qin, Zhiyong Li, Li Lin, Ruisheng Li, Zhie Fang, Yanzhong Han, Wenqing Mu, Lutong Ren, Tingting Liu, Xiaoyan Zhan, Xiaohe Xiao, Zhaofang Bai

**Affiliations:** ^1^Senior Department of Hepatology, The Fifth Medical Center of Chinese PLA General Hospital, Beijing, China; ^2^School of Pharmacy, Hunan University of Chinese Medicine, Changsha, China; ^3^China Military Institute of Chinese Materia, The Fifth Medical Centre, Chinese PLA General Hospital, Beijing, China

**Keywords:** schisandrin C, Curdione, hepatic fibrosis, oxidative stress, TGF-β1/Smads signaling pathway

## Abstract

Hepatic fibrosis is the final pathway of several chronic liver diseases, which is characterized by the accumulation of extracellular matrix due to chronic hepatocyte damage. Activation of hepatic stellate cells and oxidative stress (OS) play an important role in mediating liver damage and initiating hepatic fibrosis. Hence, hepatic fibrosis can be reversed by inhibiting multiple channels such as oxidative stress, liver cell damage, or activation of hepatic stellate cells. Liuwei Wuling Tablets is a traditional Chinese medicine formula with the effect of anti- hepatic fibrosis, but the composition and mechanism of reversing hepatic fibrosis are still unclear. Our study demonstrated that one of the main active components of the Chinese medicine Schisandra chinensis, schisandrin C (Sin C), significantly inhibited oxidative stress and prevented hepatocyte injury. Meanwhile one of the main active components of the Chinese medicine Curdione inhibited hepatic stellate cell activation by targeting the TGF-β1/Smads signaling pathway. The further *in vivo* experiments showed that Sin C, Curdione and the combination of both have the effect of reversing liver fibrosis in mice, and the combined effect of inhibiting hepatic fibrosis is superior to treatment with Sin C or Curdione alone. Our study provides a potential candidate for multi-molecular or multi-pathway combination therapies for the treatment of hepatic fibrosis and demonstrates that combined pharmacotherapy holds great promise in the prevention and treatment of hepatic fibrosis.

## Introduction

Hepatic fibrosis is a compensatory pathological process in which abnormal proliferation of connective tissue in the liver occurs when the liver is injured by various chronic inflammations, which leads to excessive deposition of extracellular matrix (ECM) (Sánchez-Valle et al., 2012). It is the common pathological basis of a variety of chronic liver diseases, including chronic viral hepatitis, alcoholic steatohepatitis, non-alcoholic steatohepatitis and drug-induced liver injury (Cordero-Espinoza and Huch, 2018). It will gradually develop into liver cirrhosis and even hepatocellular carcinoma if without timely intervention (Huang et al., 2017; Cordero-Espinoza and Huch, 2018; Pan et al., 2020). Therefore, reversal of hepatic fibrosis is significant for the prevention and the treatment of cirrhosis and liver diseases. Thus far, several potential chemotherapeutic and biological agents have been developed for the intervention of hepatic fibrosis. However, there remains a lack of effective anti-hepatic fibrosis drugs in the clinic (Fagone et al., 2016; Schuppan et al., 2018).

The activation of hepatic stellate cells (HSC) is a central part of hepatic fibrosis. After hepatocytes are invaded by various inflammatory factors, cytokines such as transforming growth factor-β1 (TGF-β1) secreted by hepatocytes, immune cells, and platelets stimulate the sustained proliferation and chemotaxis of HSC (Elpek, 2014; Kumar et al., 2017; Xu et al., 2020). TGF-β/Smad is the main signal transduction pathway of liver fibrosis (Xu et al., 2016). TGF-β1 plays a key role in the process of HSC activation and proliferation. It promotes the up-regulation of pathway related proteins α-SMA and Smad3 by regulating the Smads signaling pathway, and secretes a large amount of ECM, leading to the development of fibrosis (Nagase et al., 2006; Wu et al., 2016; Mu et al., 2018). Multiple small molecules targeting TGF-β1 have been found to have the potential to prevent and treat hepatic fibrosis (Fagone et al., 2016). Some studies have demonstrated that small molecule combination or combined pharmacotherapy is a potential strategy for the prevention and treatment of hepatic fibrosis, which has been successfully used to treat hepatic fibrosis in animal models (Yang et al., 2012; Sung et al., 2018). The occurrence of oxidative stress has been detected in almost all clinical and experimental settings of chronic liver disease, and oxidative stress plays an important role in generating liver injury and initiating hepatic fibrosis by producing mitochondrial reactive oxygen species (ROS) (Parola and Robino, 2001; Sánchez-Valle et al., 2012). Reactive oxygen species-mediated hepatic fibrosis is due to the excessive production of ROS in the liver, which leads to peroxidative damage of hepatocytes (Gandhi, 2012; Islam et al., 2017; Sun et al., 2018). In addition, there is evidence show that some antioxidants have a therapeutic effect on hepatic fibrosis or cirrhosis (Guimarães et al., 2006).

In recent years, Traditional Chinese Medicine (TCM) formulations have shown unique advantages in anti-hepatic fibrosis. For example, Fuzheng Huayu Capsules (FZHY), Biejia Ruangan Tablets (BJRG), and other TCM compound preparations have been approved to treat hepatic fibrosis in the clinic by China Food and Drug Administration (Guo et al., 2004; Zhang and Schuppan, 2014). Besides, studies have confirmed that the active compound polyphenol rosmarinic acid combined with baicalin in the herbal prescription Yang-Gan-Wan has the effect of treating hepatic fibrosis (Yang et al., 2012). Liuweiwuling tablets (LWWL) is a TCM formula approved by the Chinese State Food and Drug Administration (CFDA) to reduce transaminase levels caused by liver disease with chronic hepatitis B. And which is composed of six herbs, namely Schisandra, Ligustrum lucidum, Forsythia, Zedoary Turmeric, Perennial Sow Thistle, and Ganoderma lucidum spores (Liu et al., 2018a, b; Ai et al., 2021). Our previous studies have found that LWWL can inhibit the activation of HSC by regulating nuclear factor kappa-B (NF-κB) and TGF-β/Smad signaling pathways, and exert its protective effect on hepatic fibrosis induced by carbon tetrachloride (CCl_4_) and bile duct ligation (BDL) in rats (Liu et al., 2018b; Sun et al., 2018). In addition, the bioactive lignan can inhibit the proliferation of HSC-T6 cells, and the monomeric component schisandrin B (Sin B) has been shown block HSC activation by inhibiting TGF-β/Smad signaling pathway and reduce hepatic fibrosis progress in rats (Huang et al., 2011; Chen et al., 2013, 2017).

Schisandrin C (Sin C), one of the lignans of Schisandra chinensis, has been proven to resist cholestatic liver injury caused by lithocholic acid (LCA) in mice by stimulating pregnane X receptor (PXR) and to affect the antioxidant enzyme activity in rat liver microsomes to exert antioxidant effects (Lu and Liu, 1991; Fan et al., 2019). In recent years, it has been found that Sin C may be the main active substance of LWWL against hepatic fibrosis *in vivo* (Liu et al., 2018a, b). Curdione is a sesquiterpenoid component isolated from Curcuma zedoaria (Berg.) Rosc, which has the potential to inhibit the proliferation of hepatic fibroblasts. Curdione has been reported to improve pulmonary fibrosis by inhibiting the activation of the TGF-β pathway, but experimental studies of this component against hepatic fibrosis have not been reported in the literature (Park et al., 2005; Liu et al., 2020). In this study, we proved that the combination of Curdione and Sin C synergistically reverse hepatic fibrosis via modulating the TGF-β pathway and inhibiting oxidative stress, suggesting regulating multiple signal transduction pathways to combined pharmacotherapy is a valid strategy for the treatment of hepatic. And the combination of Curdione and Sin C may be used as a potential therapeutic drug for the hepatic fibrosis.

## Experimental

### Mice

SPF class C57BL/6 mice (Male) were purchased from Beijing SPF Biotechnology Co., Ltd. All mice were used at approximately weighting 18–20 g. The animals were housed at Fifth Medical Centre, Chinese PLA General Hospital for 1 week prior to the initiation of this study. All animal care and experimental procedures in this study were performed in accordance with the guidelines for care and use of laboratory animals of Fifth Medical Centre, Chinese PLA General Hospital. This study was reviewed and approved by the animal ethics committee of the Fifth Medical Centre, Chinese PLA General Hospital (Beijing, China).

### Reagents and Chemicals

Schisandrin A, Schisandrin B, Schisandrin C (SinC), Schisandrol B, Isocurcumenol, Curdione, Curcumenol, Specnuezhenide, Salidroside, Phillyrin, Esculetin, Apigenin, and SYBR Green Mix were purchased from MedChemExpress (NJ, United States). Anti-Collagen1 (1:500), anti-mouse-Smad2/3 (1:2,000), and anti-mouse-P-Smad3 (1:1,000) primary and horseradish peroxidase-conjugated secondary antibodies (1:5,000) were purchased from Cell Signaling Technology (Boston, United States). The mouse Collagen IV enzyme-linked immunosorbent assay (ELISA) kits and anti-human Smad3 (1:500), anti-human P-Smad3 (1:2,000) were purchased from Abcam (Cambridge, United Kingdom). Anti-human α-SMA (1:1,000) was purchased from ABclonal (Wuhan, China) and anti-GAPDH (1:5,000) was obtained from Proteintech (Chicago, United States). The kits for determining alanine aminotransferase (ALT), aspartate aminotransferase (AST) activity, total bilirubin (TBIL), and direct bilirubin (DBIL) content were obtained from Jiancheng (Nanjing, China). Malondialdehyde (MDA) and glutathione (GSH) test kits were supplied by KANGLANG (Shanghai, China). Lactate Dehydrogenase (LDH) cytotoxicity assay kit was purchased from Beyotime (Shanghai, China). Mouse tissue hydroxyproline (Hyp) levels were measured by enzyme-linked immunosorbent assay (ELISA) kits (Cloud-Clone Corp, Houston, TX, United States). MitoSOX^TM^ Red mitochondrial superoxide indicator was manufactured by Invitrogen (Carlsbad, CA, United States). Enhanced chemiluminescent reagents was purchased from Millipore (Beijing, MA, United States).

### Animal Experiment Protocol

This experiment was divided into the following groups, including control group (Control), model group (MCD feed), positive drug (colchicine) group (Col, 0.2 mg/kg), Sin C administration group alone (200 mg/kg), Cur administration group alone (50 mg/kg), Sin C + Cur combined administration group (200 + 50 mg/kg). The experimental modeling lasted for 6 weeks. After the C57BL/6 mice were adaptively reared for 1 week, used MCD feed to conduct an experimental model. Except for the normal control group, the other groups were given the solvent and administered according to the above-mentioned protocol for 6 weeks.

### Serum Biochemistry

Blood samples were centrifugated at 1,500 *g* for 15 min to separate serum. The serum ALT and AST levels, TBIL and DBIL contents were measured using commercial kits.

### Enzyme-Linked Immunosorbent Assay

Mouse liver samples were homogenized with extraction buffer to extract the proteins, then each sample was normalized by the total protein concentration. Then the levels of Collagen IV and hydroxyprolinein liver were determined using the assay kits in accordance with the manufacturer’s instructions. Supernatants from cell culture were assayed with MDA according to manufacturer’s instructions.

### Cell Culture and Treatment

LX-2 and L0-2 cells were cultured in RPMI 1640 and Dulbecco’s modified eagle medium (DMEM) medium containing 10% fetal bovine serum (FBS) and 1% penicillin/streptomycin (P/S), respectively. All cells were cultured in a sterile cell culture incubator at 37°C with 5% CO_2_.

### CCK-8 Assay for Cell Viability

L02 and LX-2 cells were seeded overnight in 96-well plates at a density of 1.5 × 10^4^ and 2.5 × 10^4^ cells/well, respectively, and treated with SinC and Curdione (0–120 μM) for 24 h. Subsequently, the cell counting kit-8 (CCK-8) working solution was added to the cells according to the manufacturer’s instructions and incubated at 37°C for 30 min. Measure the absorbance at 450 nm with a microplate reader (BioTek, VT, United States).

### Lactate Dehydrogenase Assay

L02 cells were seeded in a 24-well plate overnight at a density of 1.5 × 10^4^ cells/well and the cells were treated with SinC for 6 h, and exposed to APAP (20 mM) for 12 h. Then LDH release was determined using the LDH Assay according to the manufacturer’s instructions.

### Measurement of Cellular Reactive Oxygen Species

The L02 cells were pretreated with Schisandrin A (40 μM), Schisandrin B (40 μM), Schisandrin C (SinC) (40 μM), Schisandrol B (40 μM), Isocurcumenol (40 μM), Curdione (40 μM), Curcumenol (40 μM), Specnuezhenide (40 μM), Salidroside (40 μM), Phillyrin (40 μM), Esculetin (40 μM), Apigenin (40 μM) for 6 h and exposed to APAP (20 mM) for 12 h. Next, the ROS levels were determined according to the previous description with some modifications (Shi et al., 2020). In addition, the determination of ROS generation with different concentrations of Sin C (10, 20, and 40 μM) in the presence or absence of APAP as described in the previous step.

### Cell Culture Treatments and Sample Collection

LX-2 cells were seeded in a 24-well plate at a concentration of 7.5 × 10^4^ cells/well and placed in the incubator overnight. The cells were treated with Curdione for 1 h, and exposed to TGF-β1 (10 ng/ml) for 24 h. The supernatant was then discarded and 1 × Laemmli sample loading buffer or TRIZOL (Invitrogen) was added to lyse the cells and the samples were collected for downstream experiments.

### Western Blotting

The expressions of Collagen I, P-Smad3, Smad3, and α-SMA in mice liver tissue and LX-2 cells were analyzed by Immunoblot performed as described previously (Ai et al., 2021).

### Real-Time Quantitative Polymerase Chain Reaction

Quantitative Polymerase Chain Reaction (Q-PCR) analysis was used to determine gene expression in LX-2 cells and mouse liver tissue from each experimental group 25. TRIZOL (Invitrogen) reagent was used to extract total RNA from mouse liver tissue and LX-2 cells, according to the supplier’s instruction. RT Master Mix for qPCR was employed based on the manufacturer’s instructions for the amplification of the target gene. Use the instrument to amplify each target gene following previous methods. Primer sequences are shown in Table 1.

**TABLE 1:** Primer sequences for Real−time quantitative polymerase chain reaction.

Target gene		Sequence (5′–3′)
mouse Acta2	Forward	CCCAGACATCAGGGAGTAATGG
	Reverse	TCTATCGGATACTTCAGCGTCA
mouse Collagen I	Forward	CTGGCGGTTCAGGTCCAAT
	Reverse	TTCCAGGCAATCCACGAGC
mouse GAPDH	Forward	TGGCCTTCCGTGTTCCTAC
	Reverse	GAGTTGCTGTTGAAGTCGCA
mouse Smad3	Forward	TCTCCCCGAATCCGATGTCC
	Reverse	GCTGGTTCAGCTCGTAGTAGG
mouse TGF-β1	Forward	CTTCAATACGTCAGACATTCGGG
	Reverse	GTAACGCCAGGAATTGTTGCTA
human Acta2	Forward	CAGGGCTGTTTTCCCATCCAT
	Reverse	GCCATGTTCTATCGGGTACTTC
human Collagen I	Forward	GTCGAGGGCCAAGACGAAG
	Reverse	CAGATCACGTCATCGCACAAC
human Smad3	Forward	CCATCTCCTACTACGAGCTGAA
	Reverse	CACTGCTGCATTCCTGTTGAC
human GAPDH	Forward	AGCCACATCGCTCAGACAC
	Reverse	GCCCAATACGACCAAATCC

### Statistical Analyses

Prism 6 (GraphPad Software, CA, United States) software was used for statistical analysis, and the experimental data were presented as mean ± standard deviation (Mean ± SD). One way ANOVA followed by an unpaired Student’s *t*-test or Dunnett’s multiple comparison *post hoc* test were used, and the difference was statistically significant with *P* < 0.05.

## Results

### Numerous Drugs Have the Ability to Inhabit Acetaminophen-Induced Oxidative Stress and TGF-β1/Smad-Mediated Activation of Hepatic Stellate Cells *in vitro*

**FIGURE 1:**
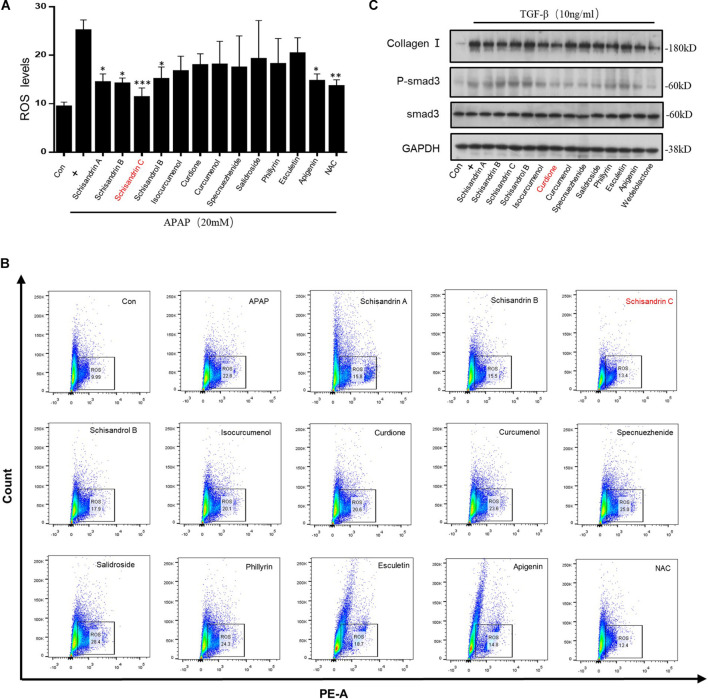
Screening of active ingredients *in vitro* based on inhibiting oxidative stress and blocking TGF-β1/Smad-mediated hepatic stellate cells (HSC) activation. **(A,B)** The percentage of ROS positive cells were detected with MitoSox staining and analyzed by FACS. **(C)** Western blot analysis of Collagen I, Smad3, p-Smad3, GAPDH in LX-2 cells treated with twelve active ingredients (40 μM) in Liuwei Wuling tablets (LWWL) and then stimulated with TGF-β1 (10 ng/ml). Data are expressed as the mean ± SD of three independent experiments in panels **(A,B)**, *p < 0.05, **p < 0.01, and ***p < 0.001.

The composition and mechanism of LWWL reversal of hepatic fibrosis are not yet clear. Persistent hepatocyte damage is the fundamental factor of fibrosis. The accumulation of ROS in cells will cause cell oxidative damage. It is also one of the important causes of fibrosis (Islam et al., 2017). Hence, it can be achieved by inhibiting the accumulation of ROS, reduce hepatocyte damage to reverse hepatic fibrosis. To further evaluate the effect of LWWL on hepatic fibrosis, twelve active constituents in the LWWL [schisandrin A, schisandrin B, schisandrin C (Sin C), schisandrol B, isocurcumenol, curdione, curcumenol, specnuezhenide, salidroside, phillyrin, esculetin, and apigenin] were chosen for testing. Acetaminophen (APAP) is a commonly used antipyretic and analgesic drug. Excessive use can cause oxidative damage to hepatocytes (Yan et al., 2018). Acetaminophen pretreatment on L02 cells will cause the accumulation of ROS. Hence, APAP-primed L02 cells were treated with twelve drugs for 6 h. The results showed that Schisandrin A, Schisandrin B, Schisandrin C, and Esculetin could inhibit the accumulation of ROS caused by APAP as the same as nacetylcysteine (NAC). Nacetylcysteine as the precursor of GSH is the only antidote for APAP overdose, which can significantly inhibit the accumulation of ROS caused by APAP (Corcoran et al., 1985; Corcoran and Wong, 1986). Among them, SinC showed the most potent effect on inhibiting the accumulation of ROS (Figures 1A,B). Moreover, The TGF-β1/Smad pathway plays a key role in the hepatic fibrosis pathway (Xu et al., 2016). Hence, we used TGF-β1 stimulation to activate the TGF-β1/Smad signaling pathway, and then pretreated with twelve drugs to observe the effects of these drugs on the TGF-β1/Smad signaling pathway. The result showed that Curdione had the strongest effect (Figure 1C). Therefore, we next separately focused on investigating the effect of Sin C on oxidative stress, the influence of Curdione on the TGF-β1/Smad signaling pathway and the role of the combination of two components in hepatic fibrosis.

### Schisandrin C Inhibits Acetaminophen-Induced Oxidative Stress *in vitro* in a Dose-Dependent Manner

**FIGURE 2:**
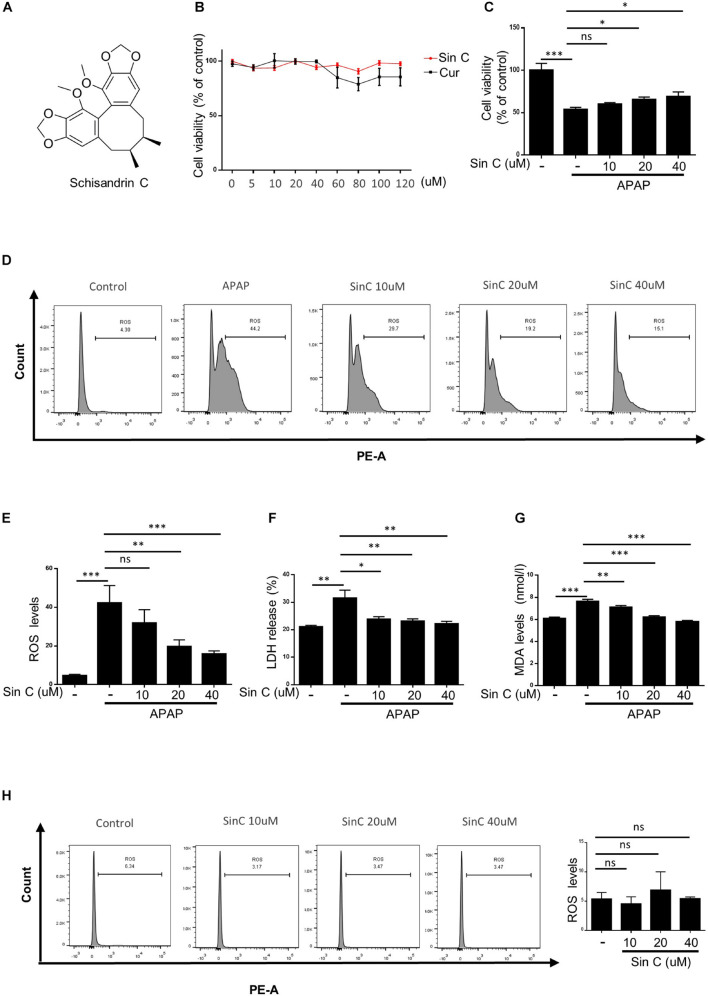
Schisandrin (Sin C) inhibits acetaminophen (APAP)-induced oxidative stress *in vitro*. **(A)** Structure of Sin C. **(B)** The Cell viability of L02 cells treated with different concentrations of Sin C and Curdione was determined using CCK-8 kit. **(C–G)** L02 cells were pretreated with SinC (10, 20, and 40 μM) for 6 h and then exposed to APAP (20 mM) for a further 12 h. The survival rate by CCK-8 **(C)** and the release of LDH **(D)** and MDA **(E)** were detected. **(F–G)** The percentage of reactive oxygen species (ROS) positive cells were detected with MitoSox staining and analyzed by FACS. **(H)** L02 cells were treated with SinC (10, 20, and 40 μM) for 18 h without any treatment and the percentage of ROS positive cells were detected with MitoSox staining and analyzed by FACS. Data are expressed as the mean ± SD of three independent experiments, *p < 0.05, **p < 0.01, and ***p < 0.001; ns, not significant.

The chemical structure of Sin C is shown in Figure 2A. we treated APAP primed L02 cells with the twelve drugs for 24 h. CCK-8 assay results showed that cell viability was not affected by Sin C or Curdione treatment compared with control cells (at a concentration of 0–120 μM) (Figure 2B), indicating that Sin C and Curdione had no significant toxic effects on L02 cells within the effective concentration range. And then, we examined whether Sin C suppressed hepatocyte damage in an effective concentration range (0, 10, 20, and 40 μM). At the same time, the ability of Sin C to eliminate intracellular ROS on hepatocytes was further examined by using the MitoSOX^TM^ Mitochondrial Superoxide Red Fluorescent Probe. The results showed that Sin C treatment increased cell viability in a dose-dependent manner and inhibited the accumulation of ROS in L02 cells (Figures 2C–G). In addition, Sin C also inhibited the release of LDH and the production of MDA in the cell supernatant in the presence of APAP. After in the same conditions, L02 cells were treated with Sin C only, the result showed the accumulation of ROS *in vivo* was not affected (Figure 2H).

### Curdione Prevents Hepatic Stellate Cell Activation *in vitro* Mediated by TGF-β1/Smad Signaling Pathway

**FIGURE 3:**
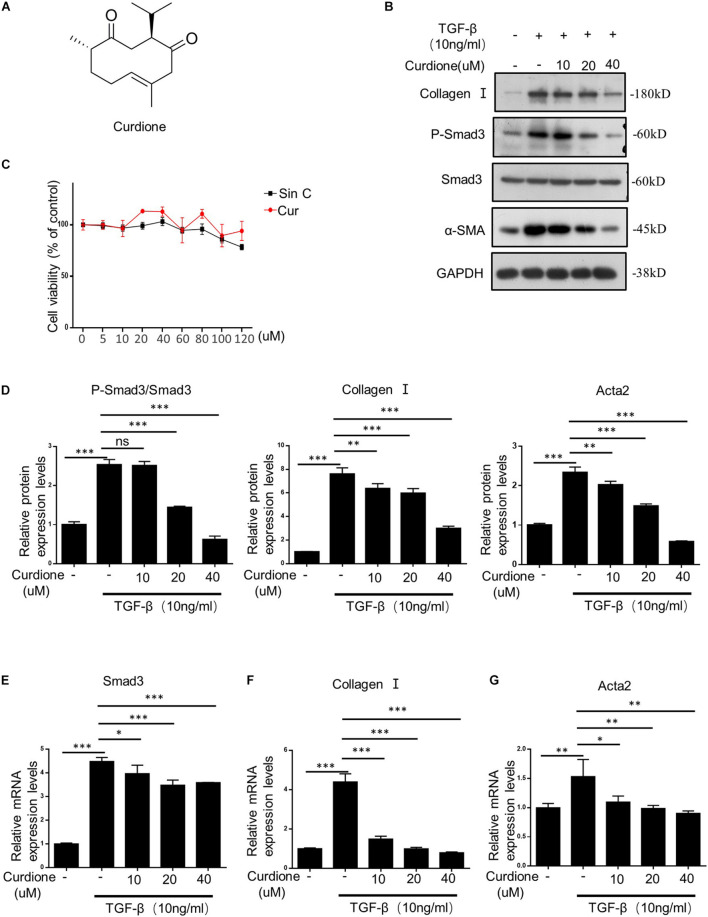
Curdione inhibits TGF-β1/Smad-mediated activation of hepatic stellate cells (HSCs). **(A)** Structure of Curdione. **(C)** The Cell viability of LX-2 cells treated with different concentrations of SinC and Curdione was determined using CCK-8 kit. LX-2 cells treated with Curdione (10, 20, and 40 μM) and then stimulated with TGF-β1 (10 ng/ml), the protein of Collagen I, α-SMA, Smad3, p-Smad3, GAPDH were detected by Western blot analysis **(B,D)**. And quantitative PCR analysis of Collagen I, α-SMA, Smad3 mRNA levels **(E–G)**. Data are expressed as the mean ± SD of three independent experiments in panels **(C–G)**, *p < 0.05, **p < 0.01, and ***p < 0.001; ns, not significant.

The chemical structure of Curdione is shown in Figure 3A. The TGF-β1/Smad signaling pathway mediates the continuous synthesis of collagen in activating HSCs and plays a central role in the pathogenesis of hepatic fibrosis (Mu et al., 2018). Our results showed that cell viability was not affected by Curdione treatment for 24 h compared with control cells (at a concentration of 0–120 μM Curdione) (Figure 3C), indicating that Curdione had no significant toxic effects on LX-2 cells within the effective concentration range. Based on these results, we investigated whether Curdione had an effect on inhibiting TGF-β1/Smad signaling *in vitro*. LX-2 cells were pretreated with Curdione (0, 10, 20, and 40 μM) for 1 h and then exposed to TGF-β1 (10 ng/ml) for a further 24 h. Our results showed that there was a decrease in expression and phosphorylation of Smad3 in the TGF-β1 signaling after Curdione pretreatment (Figures 3B,D). Curdione also down-regulated α-SMA and collagen I expression mediated by TGF-β1 in a dose-dependent manner (Figures 3B,D). Similarly, Curdione inhibited the mRNA levels of Smad3, α-SMA and collagen I (Figures 3E–G). Combining the above results, we reveal that Curdione suppresses the activation of HSCs by targeting the TGF-β1/Smad signaling pathway.

### The Combination of Schisandrin C and Curdione Significantly Alleviates Hepatic Fibrosis in Mice With MCD-Induced Hepatic Fibrosis

**FIGURE 4:**
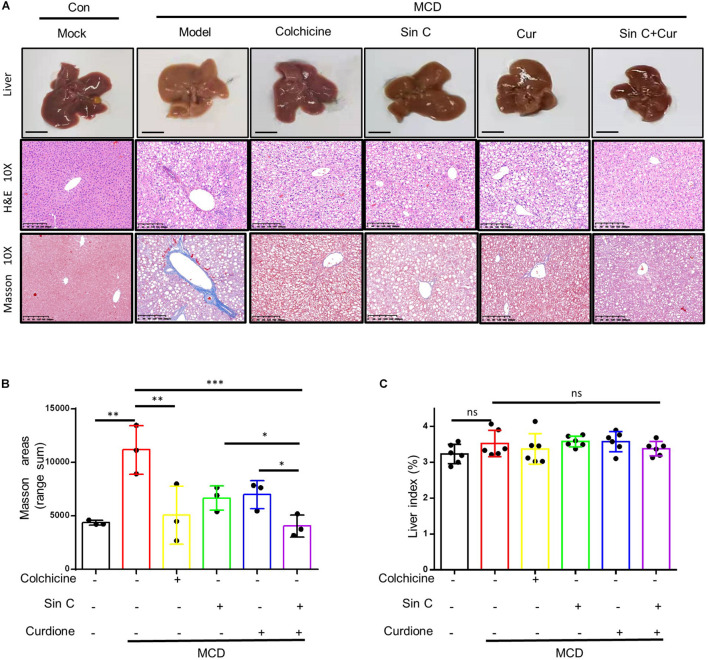
The combination of Sin C and Curdione treatment significantly improved the histopathological changes in the liver of mice induced by MCD feed. The mice were pretreated with SinC, Curdione or combination of SinC and Curdione vehicle by gavage and fed with MCD feed for 6 weeks. Histopathological changes in the liver **(A)**, Scale bars represent 200 μm Quantitative results of Masson staining sections **(B)** and liver coefficients **(C)**. Data are expressed as the mean ± SD from three independent mice samples **(A,B)**, *p < 0.05, **p < 0.01, and ***p < 0.001; ns, not significant.

**FIGURE 5:**
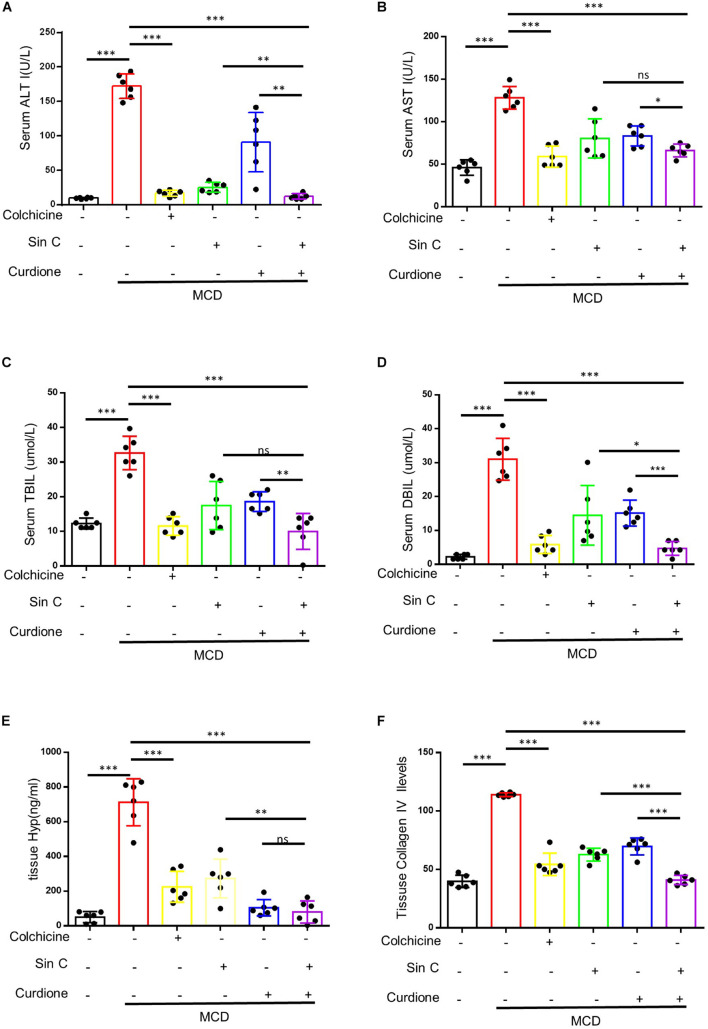
A combination of Sin C and Curdione treatment dramatically inhibits hepatic fibrosis and injury in MCD feed fed-induced mice. Mice were treated as described in Figure 4. Serum level of ALT **(A)**, AST **(B)**, TBIL **(C)**, and DBIL **(D)** were detected. The amount of hydroxyproline **(E)** and Collagen IV **(F)** in liver of mice were measured using commercial ELISA kits. Data are expressed as the mean ± SD from six independent mice samples, *p < 0.05, **p < 0.01, and ***p < 0.001; ns, not significant.

We have demonstrated *in vitro* that Sin C had the effect of resisting oxidative stress and protecting hepatocytes, and Curdione could target and regulate the TGF-β1/Smad pathway. We further evaluated whether the Sin C and Curdione combined mediated anti-hepatic fibrosis effect is better than the effect of Sin C and Curdione alone in the MCD-induced hepatic fibrosis mouse model. The MCD diet was used to induce hepatic fibrosis and the mice were randomly divided into six groups, except for the control group, all the other groups were fed with MCD fodder. Compared with control group, model group mice showed the histopathological changes of HE and Masson confirmed hepatocytes injury and hepatic fibrosis. Curdione, Sin C, and their combined treatment groups showed good effects in preventing hepatic fibrosis. Moreover, the coordinate repression of Curdione and Sin C on hepatic fibrosis was significantly superior than Curdione or Sin C alone (Figures 4A,B). Hyproxyproline is a unique amino acid component of collagen in the body. Together with Collagen IV, it serves as an important indicator of collagen metabolism in the body (Attallah et al., 2007; Mak and Mei, 2017). Consistent with the results of liver histopathological evaluation, the combination therapy of Curdione and Sin C also reduced the content of hydroxyproline and Collagen IV in liver tissue more effectively than Curdione or Sin C alone (Figures 5E,F). In addition, the serum levels of ALT, AST, DBIL, and TBIL in mice induced by MCD are consistent with histopathological analysis (Figures 5A–D).

### The Combination of Schisandrin C and Curdione Reduces MCD-Induced Hepatic Fibrosis by Regulating the TGF-β1/Smad Pathway and Inhibiting Oxidative Stress

**FIGURE 6:**
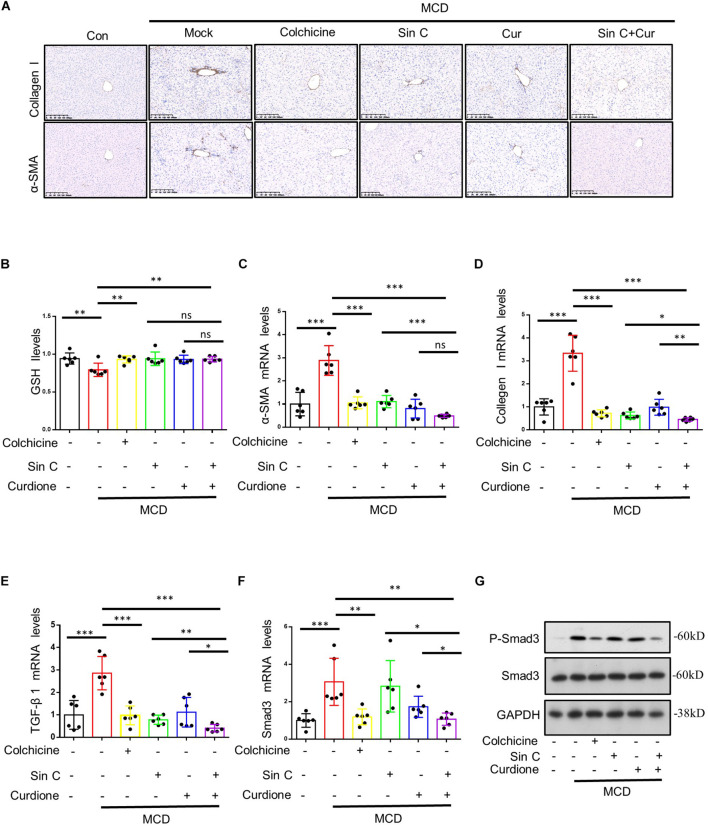
The combination of Sin C and Curdione reduces MCD-induced hepatic fibrosis by regulating the TGF-β1/Smad pathway and inhibiting oxidative stress. Immunohistochemical staining of α-SMA and collagen I **(A)** The amount of glutathione (GSH) **(B)** in liver of mice were measured using commercial ELISA kits. Quantitative polymerase chain reaction (PCR) analysis of mRNA levels of α-SMA, Collagen I, TGF-β 1, and Smad3 **(C–F)**. Western blot analysis of p-Smad3, Smad3, and GAPDH in livers from control, MCD fed-induced hepatic fibrosis mice, MCD fed-induced hepatic fibrosis mice treated with colchicine (0.2 mg/kg), Sin C (200 mg/kg), Curdione (50 mg/kg) or combination of Curdione and Sin C **(G)**. Data are expressed as the mean ± SD from six independent mice samples **(B–F)**, *p < 0.05, **p < 0.01, and ***p < 0.001; ns, not significant.

Excessive consumption of GSH is a important way to produce ROS that leads to cell oxidative damage (Wu et al., 2019). For this reason, we measured the level of GSH in liver tissue. The results showed that Curdione and Sin C alone or in combination with both increased the consumption of GSH caused by MCD to varying degrees (Figure 6B). α-SMA is a unique marker that activates HSC to cause hepatic fibrosis, so we detected the expression of α-SMA by immunohistochemical staining and qPCR (Figures 6A,C). Our immunohistochemical results showed that Curdione and Sin C treatment reduced collagen I and α-SMA expression in MCD-induced hepatic fibrosis mice (Figure 6A). As revealed by qPCR analysis (Figures 6C,D), Curdione and Sin C treatment also significantly reduced the MCD-induced increase in collagen I and α-SMA mRNA levels. The activation and continuous activation of HSC are regulated by the TGF-β1/Smad signaling pathway mediated by TGF-β1 (Inagaki and Okazaki, 2007; Dewidar et al., 2019). Therefore, we used western blotting to detect the expression of P-Smad 3/Smad 3 in liver tissue. Compared with the control group, the protein expression level of P-Smad 3/Smad 3 in the model group was significantly increased, and the protein level decreased after the administration of Curdione, Sin C, and the combination of Curdione and Sin C (Figure 6G). Moreover, the combination group was significantly better than the single administration group. Q-PCR analysis of TGF-β1 and Smad 3 mRNA level is consistent with protein expression level (Figures 6E,F).

## Discussion

Our present research demonstrates that SinC, Curdione, or the combination of SinC and Curdione can reverse hepatic fibrosis *in vivo*, evidenced by the alleviation of pathological lesions and the changes in biochemical indicators. Among them, the combined effect of SinC and Curdione in inhibiting hepatic fibrosis is better than SinC or Curdione alone. In addition, SinC can inhibit the accumulation of ROS *in vitro*, improve cell survival, and reduce the release of LDH and Curdione can block the activation of HSC by acting on TGF-β1/Smad signaling pathway. Our research confirms the combination of drugs that inhibits oxidative stress *in vivo*, reduces hepatocyte damage and blocks the TGF-β1/Smad pathway to reduce ECM deposition will more effectively solve hepatic fibrosis.

Hepatic fibrosis is secondary to liver damage (Gieling et al., 2009; Aydın and Akçalı, 2018). Hepatocyte damage can further promote the activation of HSC in the liver (Aydın and Akçalı, 2018). Therefore, reversing hepatocyte damage is one of the important ways to prevent and treat hepatic fibrosis. The production of ROS in the liver is an important cause of hepatocyte damage (Lee et al., 2015). When the liver is stimulated by foreign substances such as acetaminophen and ethanol, it will cause excessive accumulation of ROS in the liver and further aggravate hepatocyte damage (McGill and Jaeschke, 2013). In addition, the further stimulate the activation of HSC can be mediated by ROS or other secretions such as superoxide anion (Cheng et al., 2019). After HSCs are activated by ROS, they further promote the excessive secretion and accumulation of ECM, leading to hepatic fibrosis. In our research, we found that SinC can reduce the accumulation of ROS, the release of LDH and MDA in cells, and protect LO2 cells from APAP damage. In MCD-induced hepatic fibrosis model mice, it plays a superior role in protecting liver and anti-oxidation. This indicates that SinC can reverse hepatic fibrosis by inhibiting oxidative stress and reduce further damage to hepatocytes.

The key to hepatic fibrosis is the activation of HSC (Bataller and Brenner, 2005), which can mediate TGF-β1/Smad signaling pathway and cause the formation of hepatic fibrosis (Inagaki and Okazaki, 2007). Studies have shown that TGF-β1 siRNA significantly down-regulates the expression of TGF-β1, thereby inhibiting the activation and proliferation of HSCs, and reversing rat hepatic fibrosis (Hafez et al., 2017). The TGF-β1/Smad signaling pathway can enhance the activation of stellate cells and play an important role in the excessive accumulation of ECM components induced by TGF-β1 (Lang et al., 2011; Duan et al., 2014). Our data showed that the expression of p-Smad3 in LX-2 cells was up-regulated under the stimulation of TGF-β1, as well as the activated HSC markers α-SMA and collagen I. In addition, the expression of TGF-β1 and p-Smad3 in the liver tissue of mice with hepatic fibrosis induced by MCD was also up-regulated. However, Curdione partially reversed the levels of p-Smad3, α-SMA, and collagen I in LX-2 cells and mouse liver tissue in response to TGF-β1 (Zhou et al., 2017). The above data indicate that Curdione can inhibit TGF-β1-induced HSC activation by modulating TGF-β1/Smad signaling.

To date, there is no internationally recognized safe, effective and liver-targeted drug that has been approved by the FDA for the treatment of human hepatic fibrosis (Campana and Iredale, 2017), the general treatment is to eliminate chronic stress and liver transplantation. Combination therapies that target multiple pathways or involve multiple key molecules are considered to have great promise for the treatment of hepatic fibrosis (Trautwein et al., 2015). Hepatocyte damage and HSCs activation are important reasons for the development of hepatic fibrosis. SinC and Curdione can reverse hepatic fibrosis by inhibiting oxidative stress, protecting hepatocytes and regulating TGF-β1/Smad signal pathway to block HSC activation, respectively. More importantly, the two combined play a superior role in reversing hepatic fibrosis, and can be used as a promising candidate for the treatment of hepatic fibrosis.

## Data Availability Statement

The raw data supporting the conclusion of this article will be made available by the authors, without undue reservation.

## Ethics Statement

The animal study was reviewed and approved by Animal Ethics Committee of the Fifth Medical Centre, Chinese PLA General Hospital.

## Author Contributions

WD, QQ, ZL, XZ, ZB, and XX participated in research design. WD, QQ, and ZL wrote or contributed to the writing of the manuscript. WD, QQ, ZL, LL, RL, and TL conducted experiments. XZ, ZB, and XX contributed new reagents or analytic tools. YH, ZF, and LR performed data analysis. All authors contributed to the article and approved the submitted version.

## Conflict of Interest

The authors declare that the research was conducted in the absence of any commercial or financial relationships that could be construed as a potential conflict of interest.

## Publisher’s Note

All claims expressed in this article are solely those of the authors and do not necessarily represent those of their affiliated organizations, or those of the publisher, the editors and the reviewers. Any product that may be evaluated in this article, or claim that may be made by its manufacturer, is not guaranteed or endorsed by the publisher.

## Abbreviations

ALT: alanine aminotransferase
APAP: acetaminophen
AST: aspartate aminotransferase
BJRG: Biejia Ruangan tablets
CCK-8: cell counting kit-8
CFDA: Chinese State Food and Drug Administration
DBIL: direct bilirubin
ECM: extracellular matrix
ELISA: enzyme-linked immunosorbent assay
FZHY: Fuzheng Huayu capsules
GSH: glutathione
HSC: hepatic stellate cells
Hyp: hydroxyprolinein
LCA: lithocholic acid
LWWL: Liuwei Wuling tablets
MDA: malondialdehyde
NAC: nacetylcysteine
NF-κB: nuclear factor kappa-B
OS: oxidative stress
PXR: pregnane X receptor
ROS: reactive oxygen species
SinC: Schisandrin C
TBIL: total bilirubin
TCM: traditional Chinese medicine
TGF-β1: transforming growth factor- β 1.
